# The Effects of Lifestyle Interventions on (Long-Term) Weight Management, Cardiometabolic Risk and Depressive Symptoms in People with Psychotic Disorders: A Meta-Analysis

**DOI:** 10.1371/journal.pone.0112276

**Published:** 2014-12-04

**Authors:** Jojanneke Bruins, Frederike Jörg, Richard Bruggeman, Cees Slooff, Eva Corpeleijn, Marieke Pijnenborg

**Affiliations:** 1 University of Groningen, University Medical Center Groningen, University Center Psychiatry, Rob Giel Research center, Groningen, The Netherlands; 2 Friesland Mental Health Services, Research Department, Leeuwarden, Friesland, The Netherlands; 3 GGZ Drenthe, Department of Psychotic Disorders, Assen, Drenthe, The Netherlands; 4 University of Groningen, University Medical Center Groningen, Department of Epidemiology, Groningen, The Netherlands; 5 University of Groningen, Department of Clinical Psychology and Experimental Psychopathology, Groningen, The Netherlands; Medical University Innsbruck, Austria

## Abstract

**Aims:**

The aim of this study was to estimate the effects of lifestyle interventions on bodyweight and other cardiometabolic risk factors in people with psychotic disorders. Additionally, the long-term effects on body weight and the effects on depressive symptoms were examined.

**Material and Methods:**

We searched four databases for randomized controlled trials (RCTs) that compared lifestyle interventions to control conditions in patients with psychotic disorders. Lifestyle interventions were aimed at weight loss or weight gain prevention, and the study outcomes included bodyweight or metabolic parameters.

**Results:**

The search resulted in 25 RCTs -only 4 were considered high quality- showing an overall effect of lifestyle interventions on bodyweight (effect size (ES) = −0.63, p<0.0001). Lifestyle interventions were effective in both weight loss (ES = −0.52, p<0.0001) and weight-gain-prevention (ES = −0.84, p = 0.0002). There were significant long-term effects, two to six months post-intervention, for both weight-gain-prevention interventions (ES = −0.85, p = 0.0002) and weight loss studies (ES = −0.46, p = 0.02). Up to ten studies reported on cardiometabolic risk factors and showed that lifestyle interventions led to significant improvements in waist circumference, triglycerides, fasting glucose and insulin. No significant effects were found for blood pressure and cholesterol levels. Four studies reported on depressive symptoms and showed a significant effect (ES = −0.95, p = 0.05).

**Conclusion:**

Lifestyle interventions are effective in treating and preventing obesity, and in reducing cardiometabolic risk factors. However, the quality of the studies leaves much to be desired.

## Introduction

Psychosis is the psychiatric term for a state of mind in which a person suffers from delusions (false beliefs that hinder a persons’ ability to function) or hallucinations (false sensory perceptions) that are not accompanied by insight [1]. Psychotic disorders include schizophrenia, schizophreniform disorder, schizoaffective disorder and delusional disorder among others. The hallucinations and delusions can be treated effectively with different types of antipsychotic drugs. However, the use of antipsychotic drugs often coincides with metabolic side-effects, such as dyslipidemia, hyperglycemia and an increase in body weight and waist circumference [2]–[4]. Obesity is a serious problem in people with psychotic disorders. The prevalence of obesity among people with psychotic disorders is 41–50%, which is substantially higher than the 20–27% prevalence in the general population [5]. A recent study mapped the body weight of people with schizophrenia during three years of antipsychotic drug use. This study showed that 34–55% of the patients with normal weight or underweight (Body Mass Index [BMI])<25 kg/m^2^) at baseline gained ≥7% of their body weight. Among the patients who were already overweight or obese (BMI≥25 kg/m^2^) at baseline this percentage was 12–42% [6]. Since obesity is a known risk factor for cardiovascular disease, this phenomenon is of major importance for the development of comorbidities in people with psychotic disorders. The cardiovascular risk that is imposed by obesity is about four times higher in people with psychotic disorders than in the general population [7]. Cardiovascular disease is one of the major causes for premature mortality in these patients [7], [8]. Not only weight gain but also increased waist circumference, high blood pressure, higher levels of triglycerides, high cholesterol and higher levels of fasting glucose and insulin contribute to the risk of cardiovascular disease and premature mortality [7], [9]. Clinical guidelines emphasize the importance of health monitoring in these patients and recommend at least annual check-ups, but they offer no recommendations with regard to lifestyle interventions [10]. Studies in the general population as well as studies in people with psychiatric disorders consistently suggest that healthy lifestyle interventions might decrease cardiovascular risk [11]. Interventions that include physical activity and improved nutritional habits presumably lead to weight reduction and increased cardiovascular fitness [12], [13]. Behavioral interventions aimed at weight loss seem promising as well, with two studies suggesting that these interventions could improve health outcomes associated with cardiometabolic risk [12], [14]. To date, several studies have examined the effectiveness of different lifestyle interventions in patients with psychotic disorders; numerous randomized controlled trials (RCTs) as well as a number of meta-analyses [15]–[17] and systematic reviews have been published [14], [18], [19]. The available reviews to date however show several limitations. First, the only meta-analysis that reported long-term post-intervention results [17] did not include all available studies [20]–[26]. Second, the quality of RCTs included in the available meta-analyses has not been assessed. Including low quality trials may yield biased results. Third, only two of the available meta-analyses included the effects of lifestyle interventions on cardiometabolic risk [16], [17]. The authors of these studies however did not report results on all relevant metabolic outcomes that were available, even though these are important factors with regard to comorbidities and mortality in this patient group. Fourth, two of the reviews had a limited patient sample: one only included studies in patients with diabetes [18] and one could not include any study because their inclusion criteria stated patients should be in primary care [19]. Last, none of the available meta-analyses reported on the effects of lifestyle interventions on depressive symptoms, although it has been widely recognized that patients with psychotic disorders often suffer from comorbid depressive symptoms [27]–[29], and that increased physical activity in these patients has been associated with lower levels of depression [30].

### Aims of the study

This study aims to investigate the effect sizes (ES) of lifestyle interventions on body weight and other cardiometabolic risk factors, such as waist circumference, blood pressure, blood lipids, glucose and insulin concentrations, in patients with psychotic disorders. The long-term effects of lifestyle interventions on body weight are included in the analysis as well. In addition, the effects of lifestyle interventions on depressive symptoms are investigated. Furthermore, we attempt to find effective components of the interventions, by comparing the studies with the largest ES and examine potential overlap and differences between the elements used in these successful interventions.

## Materials and Methods

### Inclusion criteria

A systematic search for all randomized controlled trials evaluating the effects of lifestyle interventions on weight management in patients with psychotic disorders was conducted until April 2014. The following electronic databases were searched: PubMed, Web of Science, PsycINFO and MEDLINE. Search terms included: *schizophrenia* or *schizophrenic* or *psychotic* or *schizoaffective disorder* or *mental disorder* or *mentally ill* or *psychiatric disorders* or *severe mental illness* or *antipsychotic* AND *lifestyle intervention* or *diet* or *physical activity* or *nutrition* or *lifestyle* or *body weight* or *weight loss* or *weight management* or *exercise* AND/OR *weight gain* or *metabolic* or *metabolic syndrome* or *diabetes* or *health* or *somatic* AND/OR *psychological intervention* or *counseling* or *directive counseling* or *cognitive behavioral treatment*. We included all randomized controlled trials (RCTs) that examined lifestyle interventions; *i.e.* interventions either targeting overweight patients in order to help them lose weight, or patients in the early stages of their illness in order to help them prevent antipsychotic induced weight gain. Interventions were considered lifestyle interventions when they had a nutritional element, physical activity and/or a psychological intervention aimed at weight loss or weight gain prevention. In eligible studies, all included subjects were diagnosed with psychotic disorders and study outcomes were either body weight or cardiometabolic risk factors (e.g. waist circumference, blood pressure, blood lipids, glucose and/or insulin). Pharmacological interventions were excluded, as were non-randomized studies and studies that did not qualify as lifestyle interventions. These selection criteria were first applied to the title. When the title did not present exclusion criteria (e.g. non-RCT, no intervention, no psychotic disorders) or was inconclusive, the abstracts of the articles were read and -where necessary- the full articles. Finally, the bibliographies of selected articles were searched for relevant references to be included in our analysis.

### Outcomes and calculations

The first outcome was mean body weight change, measured directly at the end of the intervention. An overall ES for lifestyle interventions was calculated as well as separate ES for weight loss interventions and weight gain prevention interventions. The same analyses were performed for the long-term effects. A sensitivity analysis was performed using the ‘Clinical Trials Assessment Measure for psychological treatments’ (CTAM) to assess the quality of the studies [31]. The CTAM determines the quality of a study based on sample size and recruitment method, allocation to treatment, assessment of outcome, control groups, description of treatments and analysis. The psychometric properties of the CTAM were found to be adequate [31]. Only the studies marked as high quality (CTAM≥65) were included in the sensitivity analysis. Next, we calculated the ES of lifestyle interventions on cardiometabolic risk factors and depressive symptoms. With regard to cardiometabolic risk, we examined all available metabolic parameters, which include waist circumference, systolic and diastolic blood pressure, total cholesterol, HDL-cholesterol, LDL-cholesterol, triglyceride concentrations, fasting glucose concentrations and fasting insulin concentrations. For depression we considered the mean changes on the depression scales reported in the studies.

### Data extraction

Relevant data that were extracted include a) patient characteristics [gender, age, diagnoses], b) intervention characteristics [duration, components, aim], c) study characteristics [dropouts, number of participants, blind assessments, control condition], d) means and standard deviations of baseline and endpoint or change scores of the outcome body weight, e) means and standard deviations of body weight at follow-up, f) means and standard deviations of baseline and endpoint or change scores of the components of all cardiometabolic risk factors as described above, g) means and standard deviations of baseline and endpoint or change scores of the outcome depressive symptoms.

In a meta-analysis all data is pooled and some variation in the intervention effects are to be expected. When the observed intervention effects are more different from each other than would be expected from random error (chance) alone it is called heterogeneity. Heterogeneity, characterized by the I^2^ statistic, is considered low when I^2^≤25% and high when I^2^≥75% [32].

### Meta-analytic procedure

To standardize the outcome among studies, Cohen’s *d* was used as a measure of ES [33]. It was calculated using the following equation:
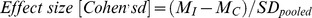



The M_I_ indicates the mean pre-post intervention difference in the intervention group, whereas M_c_ is the mean pre-post intervention difference in the control group. SD_pooled_ indicates the pooled standard deviation for both groups within one report. The Standard Error of the ES (Cohen’s *d*) was calculated by using the following equation [34]:
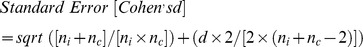



in which n_i_ and n_c_ indicate the number of participants in the intervention group and the number of participants in the control group respectively, whereas *d* stands for Cohen’s *d*. We calculated separate ES for the intervention and control group in studies that did not report the mean weight change, but only the mean weight at baseline and endpoint. Cohen’s *d* was then estimated by subtracting the ES of the control group from the ES of the intervention group. Standard Error of the ES was established by the formula mentioned above. Missing data with regard to standard deviations were imputed from the included studies. This meta-analysis was performed and written in accordance with the PRISMA-guidelines [35].

### Statistical method

The data were analyzed in RevMan Version 5.0 (Cochrane Collaboration software for meta-analyses) with the Inverse Variance method, using random effects models [36]. The χ^2^ test, based on the Q-statistic, was performed to check for the homogeneity of the effects with I^2^ as a quantifiable measure of heterogeneity [32]. Funnel plots were checked for asymmetry and Eggers’ tests were performed for each outcome to rule out publication bias [37]. Z-scores were calculated to test for overall effects.

## Results

### Study overview

**Figure 1:**
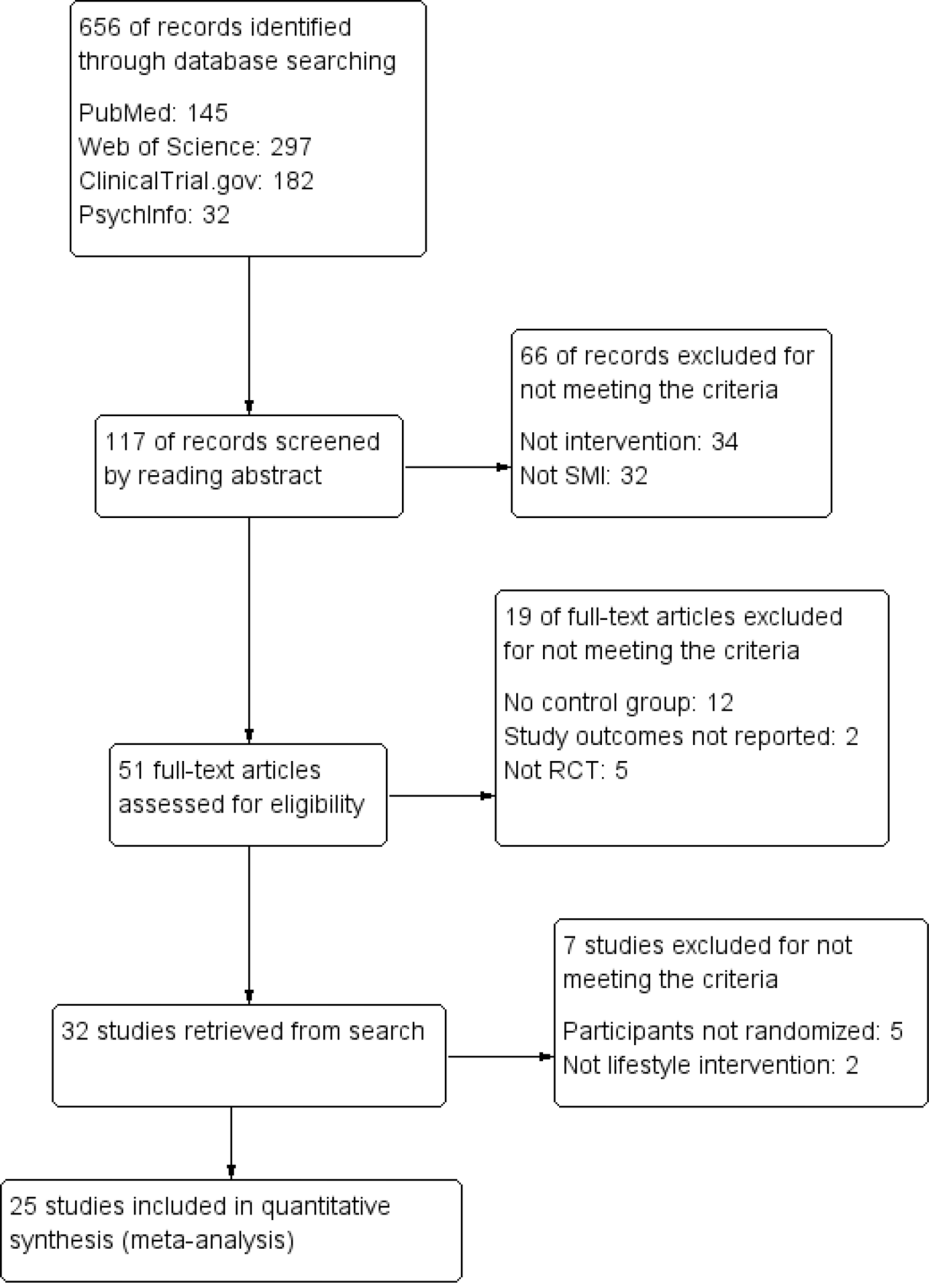
Prisma 2009 Flow Diagram.

The search resulted in 656 records in four databases. A screening of the articles and their references initially resulted in 32 eligible studies that were discussed within the research group (JB, FJ, EC, MP), which led to the exclusion of seven more studies: in five of these studies the participants were not randomly assigned to the experimental or the control group [38]–[42], and the two other studies did not meet the criteria of a lifestyle intervention [43], [44]. The remaining 25 RCT’s were included in the meta-analysis (see Figure 1). Twenty-four RCT’s reported on body weight (sixteen on weight loss and eight on weight gain prevention), seven RCT’s had long-term follow-up data on body weight (four on weight loss and three on weight gain prevention), fifteen RCT’s reported on one or more cardiometabolic risk factors and four RCT’s reported depression. Six of the studies had missing standard deviations [45]–[50], which were imputed from the data pool. All articles were published in English up to the last date of the search (April 2014).

### Study characteristics

A total of 1518 participants were included in the meta-analysis. Sample sizes across the studies varied from 14 to 291 participants with 52% male patients. The mean age across the studies varied from 26.1 (SD = 10.2) to 54.0 (SD = 9.3). Fifteen of the studies reported the use of mental health professionals, dieticians and/or exercise specialists to implement the intervention. Interventions were performed in the United States of America [22], [25], [50]–[55], South-America [21], Canada [48], the United Kingdom [45], [46], Spain [56], [57], Italy [47], [49], [58], Sweden [59], Switzerland [24], the Netherlands [60], Australia [23], Korea [61], Thailand [62], Taiwan [63] and China [64]. An overview of the study characteristics is provided in Table 1. The classification of weight loss study or weight prevention study was solely based on the aim of the researchers of the study. There were no differences with regard to the content of lifestyle interventions.

**Table 1:** Table of Characteristics.

Study ID	Sample size	Drop-outs	Participants/setting	Diagnoses	Intervention	Control condition
**Poulin et al.** **(2007)**	59 (a) 51 (b)	8%	Outpatients	Schizophrenia, schizoaffective disorder, bipolar disorder	18 month intervention with 2 group sessions per week.Supervised exercise.	Treatment As Usual
**Mauri et al.** **(2008)**	15 (a) 18 (b)	33%	Outpatients	Bipolar I and II, psychotic depression, schizoaffectivedisorder	12 week intervention, weekly 30 minute session.Diet, non-structured exercise, activity assessment andtailored advise.	Olanzapine
**Wu MK et al.** **(2007)**	28 (a) 25 (b)	5%	Inpatients	Schizophrenia	6 month intervention with 3 sessions per week. Diet andsupervised exercise.	Clozapine
**Evans et al.** **(2005)**	22 (a) 22 (b)	33%	Inpatients	Schizophrenia, schizoaffective disorder, schizophreniform,bipolar, depression	3 month intervention with 6 individual sessions of one hour.Counseling and tailored advise.	Olanzapine and passivenutrition informationby receiving a book
**Wu RR et al.** **(2008)**	32 (a) 32 (b)	8%	First psychosis	First psychotic episode of schizophrenia	12 week intervention with 10 sessions. Diet, supervised andnon-structured exercise.	Placebo
**McKibbin et al.** **(2006)**	28 (a) 29 (b)	19%	Outpatients	Schizophrenia, schizoaffective disorder and diabetesmellitus	24 week intervention with weekly group sessions.Diet encouragement and non-structured exercise.	Treatment As Usual + 3folders about diabetesmanagement
**Jean-Baptiste t al. (2007)**	8 (a) 10 (b)	22%	Outpatients	Schizophrenia, schizoaffective disorder	16 weekly group sessions. Nutritional education, goal-setting,exercise encouragement, individual advise	Treatment As Usual
**Kwon et al.** **(2006)**	33 (a) 15 (b)	25%	Outpatients	Schizophrenia, schizoaffective disorder	12 week intervention with 8 individual sessions.Nutrition and activity assessment, non-structuredexercise and tailored advise.	Treatment As Usual + dietand activity recommendation,olanzapine.
**Littrell et al.** **(2003)**	35 (a) 35 (b)	n.m.	Outpatients	Schizophrenia, schizoaffective disorder	16 week intervention with weekly group sessions.Diet encouragement, non-structured exercise and counseling.	Treatment As Usual,olanzapine
**Álvarez-Jiménez et al.** **(2006)**	28 (a) 33 (b)	0%	First psychosis	First psychotic episode	3 month intervention with 10–14 individual sessions.Diet encouragement, non-structured exercise,activity assessment, CBT and counseling.	Treatment As Usual
**Brown & Smith** **(2009)**	15 (a) 11 (b)	19%	Outpatients	Schizophrenia, major affective disorder, neurotic orpersonality disorder	5 session intervention. Nutrition and activity assessment,non-structured exercise and motivational interviewing.	Treatment As Usual
**Weber & Wyne** **(2006)**	8 (a) 7 (b)	12%	Outpatients	Schizophrenia, schizoaffective disorder	16 week intervention with weekly group sessions.Nutrition assessment, supervised exercise, CBTand counseling.	Treatment As Usual
**Methapatara t al. (2011)**	32 (a) 32 (b)	0%	Inpatients	Schizophrenia	3 month intervention with group educations,5 hourly individual sessions and practicing pedometerwalking. Non-structured exercise, motivational interviewingand counseling.	Receiving a folder abouthealthy lifestyle
**Brown & Chan** **(2006)**	15 (a) 13 (b)	39%	n.m.	Severe and enduring mental illness	6 weekly 50 minute health promotion sessions.Nutrition assessment, non-structured exercise,activity assessment, motivational interviewingand tailored advise.	Waiting list
**Daumit et al.** **(2013)**	144 (a) 147 (b)	4%	Outpatients	Schizophrenia, schizoaffective disorder, bipolar disorder,major depression, other	18 months with group and individual weight managementsessions and group supervised exercise sessions.	Standard nutrition andphysical activityinformation at baseline
**Attux et al.** **(2013)**	81 (a) 79 (b)	21%	Outpatients	Schizophrenia, other psychotic disorder	12 weekly group sessions including patients and family members,discussing diet, physical activity and stress.Food assessment with diaries.	Treatment As Usual
**Brar et al.** **(2005)**	34 (a) 37 (b)	31%	Outpatients	Schizophrenia, schizoaffective disorder	14 week interventions with 20 group sessions.Diet encouragement, nutrition assessment and CBT.	Treatment As Usual
**Skrinar et al.** **(2005)**	9 (a) 11 (b)	33%	Inpatient and outpatient	DSM IV mood- or psychotic disorder	12 week intervention with 4 hourly groupsessions per weekfor supervised exercise and 1 health seminarper week.	Waiting list
**Milano et al.** **(2007)**	22 (a) 14 (b)	n.m.	n.m.	Schizophrenia, bipolar with a manic episode	12 week intervention with 3 sessions perweek of 30–60 minutes.Diet and supervised exercise.	Olanzapine
**Khazaal et al.** **(2007)**	31 (a) 30 (b)	13%	n.m.	Receiving antipsychotic treatment	12 week intervention with weekly groupsessions.Diet encouragement, nutrition assessment, non-structured exercise,motivational interviewing and CBT.	One two hour groupeducation on healthyfood and dietrecommendation
**Brown et al.** **(2011)**	47 (a) 42 (b)	35%	n.m.	Serious mental illness	12 month intervention with 3 monthintensive, 3 month maintenance and 6 monthintermittent support phase.Diet, supervised exercise and counseling.	Treatment As Usual
**Forsberg et al.** **(2008)**	24 (a) 17 (b)	11%	Supported housing facilities	Psychiatric diagnosis DSM IV	12 month intervention, once a week cookingand once a weeksupervised exercise.	Aesthetic study(learning various artistictechniques)
**Iglesias-García et al.** **(2010)**	7 (a) 7 (b)	7%	Outpatients	Schizophrenia	3 month intervention with 12 hourly educational group sessions.Counseling.	Treatment As Usual
**Scocco et al.** **(2005)**	10 (a) 10 (b)	10%	n.m.	Schizophrenia, schizoaffective disorder	8 week intervention with weekly individualvisits to a psychiatristand nutritionist. Diet, non-structuredexercise, activity assessmentand tailored advise.	Olanzapine
**Scheewe et al.** **(2013)**	29 (a) 25 (b)	17%	n.m.	Schizophrenia, schizoaffective or schizophreniformdisorder	6 month intervention, two hours a week exercise under supervision,and six times a week muscle strength exercises	Occupational therapy(reading, painting,computer games)

*(a) Number of patients in the intervention group. (b) Number of patients in the control group. N.m. = not mentioned.* ***Structured diet*** *: prescribed diet, specific instructions regarding food- and/or calorie-intake.* ***Non-structured diet encouragement*** *: informing patients about healthy food, provide healthy food suggestions, cooking healthy meals, reimbursing purchase of healthy food and encourage healthy eating behavior without structured restrictions.* ***Nutrition assessment*** *: food diary or discussing food intake with nutritionist.* ***Supervised exercise*** *: exercising under supervision of a (personal) trainer.* ***Non-structured exercise*** *: unsupervised exercise, providing opportunity to exercise (e.g. free gym-membership), encouragement to enhance frequency and intensity of physical activity.* ***Activity assessment*** *: activity diary, discussing physical activities during sessions.* ***Psychological interventions*** *: motivational interviewing, counseling and goal-setting, cognitive-behavioral therapy (CBT) and individually tailored advice.*

### Overall results for interventions on body weight

**Figure 2:**
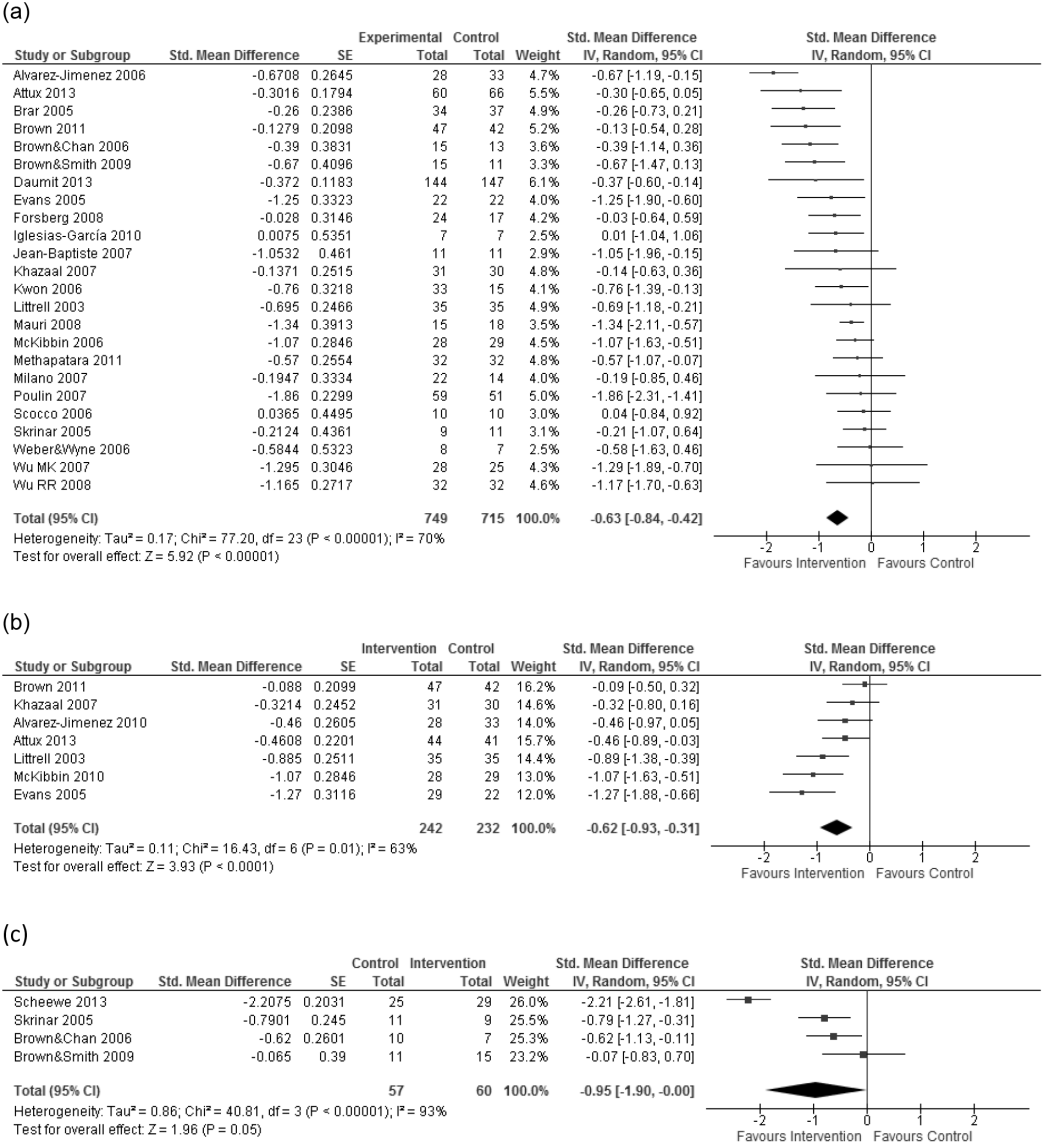
Forest plot a) describes the effects of lifestyle interventions on bodyweight. Forest plot b) describes the longterm effects of lifestyle interventions on body weight. Forest plot c) describes the effects of lifestyle interventions on depressive symptoms.

The overall ES of lifestyle interventions on weight was −0.63 (95% CI = −0.84 to −0.42). Lifestyle interventions had a significant, beneficial effect on weight loss (p<0.00001) (see Figure 2a). The experimental groups in the weight loss intervention studies showed a higher reduction of the mean body weight than the control groups (ES = −0.52 with the 95% CI = −0.72 to −0.31, p<0.00001) (see Figure S1a). In the weight gain prevention studies the experimental groups gained less body weight than the control groups (ES = −0.84 with the 95% CI = −1.28 to −0.40, p = 0.0002) (see Figure S1b). The included studies in this meta-analysis showed moderate heterogeneity. The overall intervention effect had an I^2^ of 70%. Among the weight loss intervention studies I^2^ was 55% while the I^2^ was 76% in the weight gain prevention studies.

### Study quality assessment

First, the funnel plots did not show asymmetry, but the Egger’s tests showed that publication bias could not be ruled out for diastolic blood pressure (p = 0.078) and long-term body weight (p = 0.027). All other outcomes had p-values above the recommended 0.1 on the Egger’s test, varying from 0.185 to 0.961 (see Table S1) [37]. All studies report randomization, but only 10 studies describe the randomization process [21], [22], [45], [46], [52], [56], [57], [59], [62], [64]. Five studies report the use of assessors independent from the study [55]–[57], [63], [64]; assessors were reported to be blind to group allocation in nine studies [21], [45], [46], [48], [52], [55]–[57], [64]. One study failed to adequately describe the treatment [57]. The mean drop-out rate among the studies was 17%, varying from 0–39%, but only four studies described an acceptable strategy for investigating drop-outs [51], [52], [56], [64]. Intention-to-treat analyses were reported in twelve studies [21], [45], [46], [51], [52], [56], [58]–[62], [64]. Four of the studies were considered to be of sufficient quality and had a CTAM score of at least 65 [52], [56], [59], [64]. Sensitivity analyses including these studies resulted in a decreased but still statistically significant ES of −0.55 (95% CI = −0.96 to −0.14, p = 0.008) of lifestyle interventions on body weight (see figure S2a).

### Long term effects

Seven studies reported follow up data between two and six months after the completion of the intervention [20]–[26]. Their combined ES was −0.62 (95% CI: −0.93 to −0.31, p<0.0001) as is described in Figure 2b, which is in favor of the intervention. When we analyzed weight loss and weight gain prevention studies separately, we found that interventions aimed at weight gain prevention have a large long-term effect (ES = −0.85, 95% CI = −1.29 to −0.41, p = 0.0002) (see figure S3b) while the long-term effect of the weight loss interventions was moderate (ES = −0.46, 95% CI = −0.83 to −0.08, p = 0.02) (see figure S3a). With regard to heterogeneity, I^2^ was 63% for the overall long-term effects of the intervention, 51% in the weight gain prevention studies and 62% in the weight loss studies.

### Cardiometabolic risk

Ten studies reported cardiometabolic risk factors and in these studies there were no distinct differences between the weight loss interventions and the weight gain prevention interventions with respect to the content of the intervention. Therefore, in the analyses of the metabolic parameters, the two types were taken together. The lifestyle interventions demonstrated significant effects for waist circumference, triglycerides, fasting glucose and insulin. The ES for cholesterol (total, HDL-C and LDL-C) and systolic and diastolic blood pressure were not significant. An overview is provided in Table 2. Three of the high quality studies also reported on waist circumference and insulin [52], [59], [64]. A sensitivity analysis that only included the three high quality studies resulted in slightly decreased effect sizes that were no longer statistically significant for both waist circumference (ES = −0.30, 95% CI = −0.63 to 0.03, p = 0.08) (see figure S2b) and insulin (ES = −0.26, 95% CI = −0.64 to 0.12, p = 0.18) (see figure S2c). Regarding heterogeneity, only in the studies with fasting glucose I^2^ was 0%. The remaining components had moderate to high heterogeneity with I^2^ varying from 51% to 91% (see Table 2).

**Table 2:** Effects of lifestyle interventions on cardiometabolic risk.

Parameters	N studies	N[i]*	N[c]+	Cohens *d*	95% CI	p	I^2^
Waist circumference (cm)#	10	385	320	−0.37	[−0.60; −0.13]	0.002	56%
Systolic blood pressure (mmHg)$	7	308	307	−0.22	[−0.49; 0.05]	0.10	60%
Diastolic blood pressuye (mmHg)£	3	95	76	−0.08	[−0.57; 0.41]	0.74	64%
Triglycerides (mg/dl)δ	8	338	321	−0.27	[−0.49; −0.04]	0.02	51%
HDL-cholesterol (mg/dl)Δ	8	319	308	0.28	[−0.16; 0.73]	0.21	91%
LDL-cholesterol (mg/dl)θ	5	258	259	−0.27	[−0.75; 0.22]	0.28	87%
Total cholesterol (mg/dl)§	7	295	295	−0.27	[−0.59; 0.05]	0.10	72%
Fasting glucose (mg/dl)¶	8	347	341	−0.24	[−0.32; −0.10]	0.001	0%
Insulin (µIU/ml)ψ	6	241	240	−0.28	[−0.56; −0.01]	0.04	52%

*Total N in intervention groups.

+ Total N in control groups.

# Attux et al. (2013), Daumit et al. (2013), Evans et al. (2005), Forsberg et al. (2008), Iglesias-Garcia et al. (2010), McKibbin et al. (2006), Methapatara et al. (2011), Poulin et al. (2007), Scheewe et al. (2013) & Wu RR et al. (2008).

$ Attux et al. (2013), Brar et al. (2005), Brown & Smith et al. (2009), Daumit et al. (2013), Forsberg et al. (2008), McKibbin et al. (2006) & Scheewe et al. (2013).

£ Forsberg et al. (2008), McKibbin et al. (2006) & Scheewe et al. (2013).

δ Attux et al. (2013), Daumit et al. (2013), Forsberg et al. (2008), Mauri et al. (2008), McKibbin et al. (2006), Poulin et al. (2007), Scheewe et al. (2013 &, Wu MK et al. (2007).

Δ Attux et al. (2013), Daumit et al. (2013), Forsberg et al. (2008), Mauri et al. (2008), McKibbin et al. (2006), Poulin et al. (2007), Scheewe et al. (2013) & Skrinar et al. (2005).

θ Attux et al. (2013), Daumit et al. (2013), Mauri et al. (2008), McKibbin et al. (2006) & Poulin et al. (2007).

§ Attux et al. (2013), Daumit et al. (2013), Forsberg et al. (2008), Mauri et al. (2008), McKibbin et al. (2006), Poulin et al. (2007) & Wu MK et al. (2007).

¶ Attux et al. (2013), Daumit et al. (2013), Mauri et al. (2008), McKibbin et al. (2006), Poulin et al. (2007), Scheewe et al. (2013), Wu MK et al. (2007) & Wu RR et al. (2008).

ψ Attux et al. (2013), Daumit et al. (2013), Forsberg et al. (2008), Mauri et al. (2008), Wu MK et al. (2007) & Wu RR et al. (2008).

### Depressive symptoms

Four studies reported the effects of lifestyle interventions on depressive symptoms based on a continuous depression scale. Skrinar et al. (2005) used the depression scale of the SCL-90-R, Scheewe et al. (2013) used the Montgomery Åsberg Depression Rating Scale (MADRS) and the remaining studies reported the depression score of the Hospital Anxiety and Depression Scale (HADS) [45], [46]. The overall ES for lifestyle interventions on depressive symptoms was −0.95 (95% CI = −1.90 to −0.00, p = 0.05). An overview of the results is shown in Figure 2c. None of the studies reporting depression had a CTAM score of 65 or above, so these results should interpreted with caution. Heterogeneity among the studies with outcome depression was high (I^2^ = 93%).

### Intervention characteristics

Most of the studies had an intervention period of three months or less [21], [23], [24], [45]–[47], [49], [50], [56]–[58], [61], [62], [64]. Seven studies had an intervention period between three and six months [25], [51], [53]–[55], [60], [63] and four studies had an intervention period of twelve months or more [22], [48], [52], [59]. There were also differences between the intensity and duration of the supervised exercise sessions. Exercise sessions varied from two hours per week [48], [59], [60] to 45 minutes supervised exercise four times a week [50] or daily 30 minutes sessions [22]. We studied the interventions with large effect sizes for corresponding intervention elements. However, we did not find any element present in all or most of the interventions with the largest effect sizes, nor did we find corresponding elements for the interventions with the smallest effect sizes. We did find a difference in the ES of interventions depending on whether group- or individually based interventions were used. Five studies used an individual approach in their intervention [23], [45], [49], [56], [61]. Their combined ES was −0.67 (p = 0.0004) (see figure S4a). Ten studies presented their patients with a group intervention [21], [22], [24], [25], [50], [51], [55], [57], [59], [63].The group interventions had an overall ES of −0.36 (p = 0.002) (see figure S4b). A combined approach of a group interventions accompanied by individual sessions was used in five studies [48], [52], [53], [62], [64]. They showed the largest overall ES (ES = −0.99, p = 0.002) (see figure S4c).

## Discussion

Lifestyle interventions led to weight reduction and weight gain prevention. Significant positive effects on body weight remained at follow-up. Effect sizes for weight gain prevention interventions were large and the effects of the weight loss interventions moderate. Results showed that lifestyle interventions also led to reductions in waist circumference, triglycerides, fasting glucose and insulin. No significant effects were found for blood pressure and cholesterol levels. Only four of 25 studies were of good quality. Sensitivity analyses including only these high quality studies showed a somewhat lower, but still significant, effect size for body weight. The overall effects on waist circumference and insulin were no longer significant in the sensitivity analysis. Depressive symptoms were only reported in four studies, which were of low quality. These results should thus be interpreted cautiously. In particular, in three of the four studies, the control condition consisted of a waiting list without active control treatment, indicating that non-specific effects of the intervention (e.g. extra attention, peer support) were not controlled for. Because these studies were of low quality and the effect size was on the border of significance, we cannot state with absolute certainty that lifestyle interventions effectively reduce depression.

Our findings are mostly consistent with the existing literature regarding the effects of lifestyle interventions on cardiometabolic risk in the general population. These studies found significant effects for waist circumference, fasting glucose, triglycerides [65] and insulin concentrations [66], but not one of them found a change in cholesterol levels [65]–[67]. In one study a significant effect for lifestyle interventions on systolic blood pressure was reported [67]; a finding that could not be replicated in our meta-analysis. In sum, lifestyle interventions seem to be effective in reducing most metabolic risk factors; only cholesterol and blood pressure seemed unaffected. However, the effects on waist circumference and insulin were no longer significant in the sensitivity analysis in which only the high quality studies were considered.

The characteristics of treatments were examined to provide guidelines for future clinical practice. Studies characterized by an individual approach seemed more effective than group-based interventions, while combining group treatment with individual interventions appeared to get the best result on body weight. Apparently the benefits of an individual approach, such as personal attention, advice, a tailor-made action plan and meeting patient-specific needs, surpass the benefits of group-sessions, such as group cohesiveness, interpersonal learning, imitative behavior, recognition of similarities in other group members [68], [69] and peer support [70]. That a combined approach is most effective could well be explained by the fact that these interventions encompass ‘the best of both worlds’: imitative behavior, peer support and recognition of similarities in others during group-sessions and addressing personal needs during the individual meetings.

Unfortunately, we could not identify elements specific for successful interventions. This is at odds with previous literature suggesting that diet, physical activity and psychological interventions all had an individual contribution to losing weight [12]–[14]. A previous meta-analysis [15] performed subgroup analyses to calculate effect sizes for diet, no diet, CBT, psycho-education, physical activity and no physical activity among others. However, these pooled effect sizes are difficult to interpret and may lead to unreliable conclusions since none of the interventions consisted of just one of these elements. Thus, when examining for example the effects of psychological interventions, effects of other interventions such as diet-instructions could not be filtered out, making a direct comparison of specific elements of interventions impossible.

Finally, we found that interventions based in Asia show larger effect sizes than the studies based in Central or Northern Europe, even though there were no differences between the continents with regard to the duration, intensity or elements used in the interventions or weight of the patients at the start of the intervention. This aspect has not been studies before. We find ourselves unable to explain these differences with the data currently available. Future research might elucidate these findings.

### Limitations

There was significant heterogeneity among the studies, suggesting that there were differences in the effectiveness of the lifestyle interventions that could not be a result of chance alone. Therefore, Cohen’s *d* has to be interpreted with caution. Based on the funnel plots and Egger’s test for asymmetry, we could not rule out the possibility of publication bias with regard to long term effects on body weight and the effects on diastolic blood pressure. This could indicate an overestimation of the reported effect sizes for these outcomes. Furthermore, the effects for some parameters of the metabolic syndrome and depression were based on a small number of studies. The CTAM revealed that the quality of the included studies was quite poor, with 21 out of 25 studies not meeting its standards. This questions the reliability of the effect sizes, since low quality studies tend to overestimate effects. Last, BMI change could be seen as a more meaningful outcome than weight change as it takes the height of the patients into account. Alternatively, we chose to report waist circumference and other metabolic parameters alongside body weight. Waist circumference is one of the main risk factors for cardiovascular morbidity [71]–[73]. Abdominal obesity stimulates insulin resistance, which can result in elevated triglyceride concentrations, diabetes and hypertension. All of these present an increased risk of developing cardiovascular diseases [73], [74].

### Clinical implications and future research

Lifestyle interventions in general lead to body weight loss and prevent weight gain. However, as we found no evidence for the superiority of a specific intervention model of component –other than using an individual approach- we cannot make recommendations regarding the content of lifestyle interventions. To find out which elements are more effective than others, we should test them all separately, which would be an expensive and time-consuming exercise. Also, an intervention could be more than the sum of its separate elements. It might help if future studies provided clear and detailed depictions of the content of their lifestyle interventions.

Lifestyle interventions might improve other metabolic risk factors than body weight alone, and might also improve depressive symptoms, even though only few studies reported on these outcome measures. We urge researchers investigating effects of lifestyle interventions in people with psychotic disorders to include these measures to further substantiate these findings. Given their positive effects on multiple outcomes, we recommend lifestyle interventions to be listed among other evidence based psychosocial treatments for psychosis and to be included in clinical guidelines.

The CTAM considered most of the included studies to be of poor quality. We would like to underline the importance of high quality research in order to obtain reliable results, as well as to urge researchers to properly describe the design and execution of their studies.

## Supporting Information

Figure S1 **Forest plot a) describes the effects of weight loss interventions.** Forest plot b) describes the effects of weight gain prevention interventions.
(TIF)

Figure S2 **Forest plot a) describes the sensitivity analysis of the effects of lifestyle interventions on body weight.** Forest plot b) describes the sensitivity analysis of the effects of lifestyle interventions on waist circumference. Forest plot c) describes the sensitivity analysis of the effects of lifestyle interventions on insulin.
(TIF)

Figure S3 **Forest plot a) describes the longterm follow-up effects of weight loss interventions on bodyweight.** Forest plot b) describes the longterm follow-up effects of weight gain prevention interventions on bodyweight.
(TIF)

Figure S4 **Forest plot a) describes the effects of individual interventions.** Forest plot b) describes the effects of group interventions. Forest plot c) describes the effects of combined interventions.
(TIF)

Checklist S1 **PRISMA checklist.**
(DOC)
