# Supplementary figures and images for: The Effects of Lifestyle Interventions on (Long-Term) Weight Management, Cardiometabolic Risk and Depressive Symptoms in People with Psychotic Disorders: A Meta-Analysis
Source: PLoS One. 2014 Dec 4;9(12):e112276. doi: 10.1371/journal.pone.0112276 (PMC4256304; doi:10.1371/journal.pone.0112276)

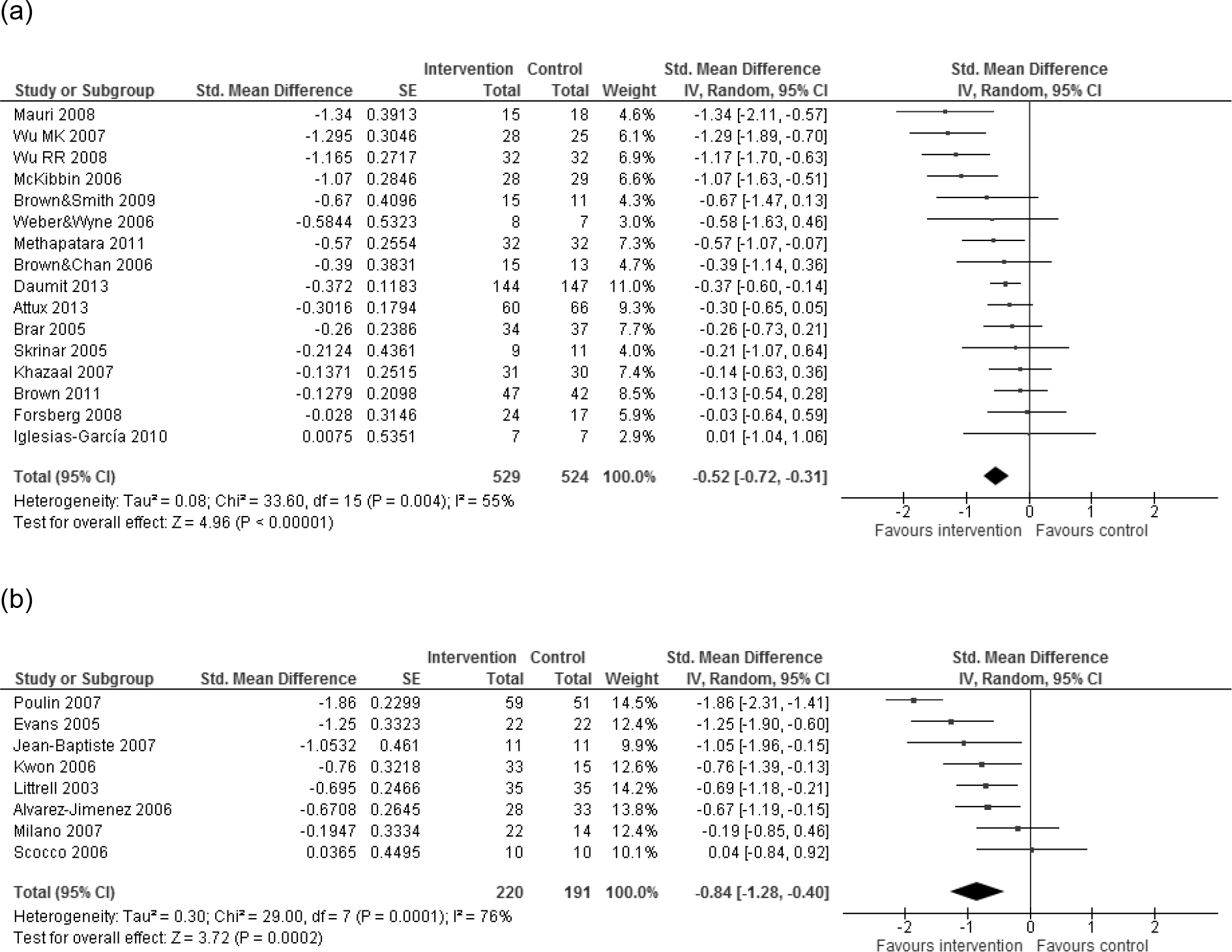

Supplement: Figure S1 — Forest plot a) describes the effects of weight loss interventions. Forest plot b) describes the effects of weight gain prevention interventions. (TIF) [file pone.0112276.s001.tif]

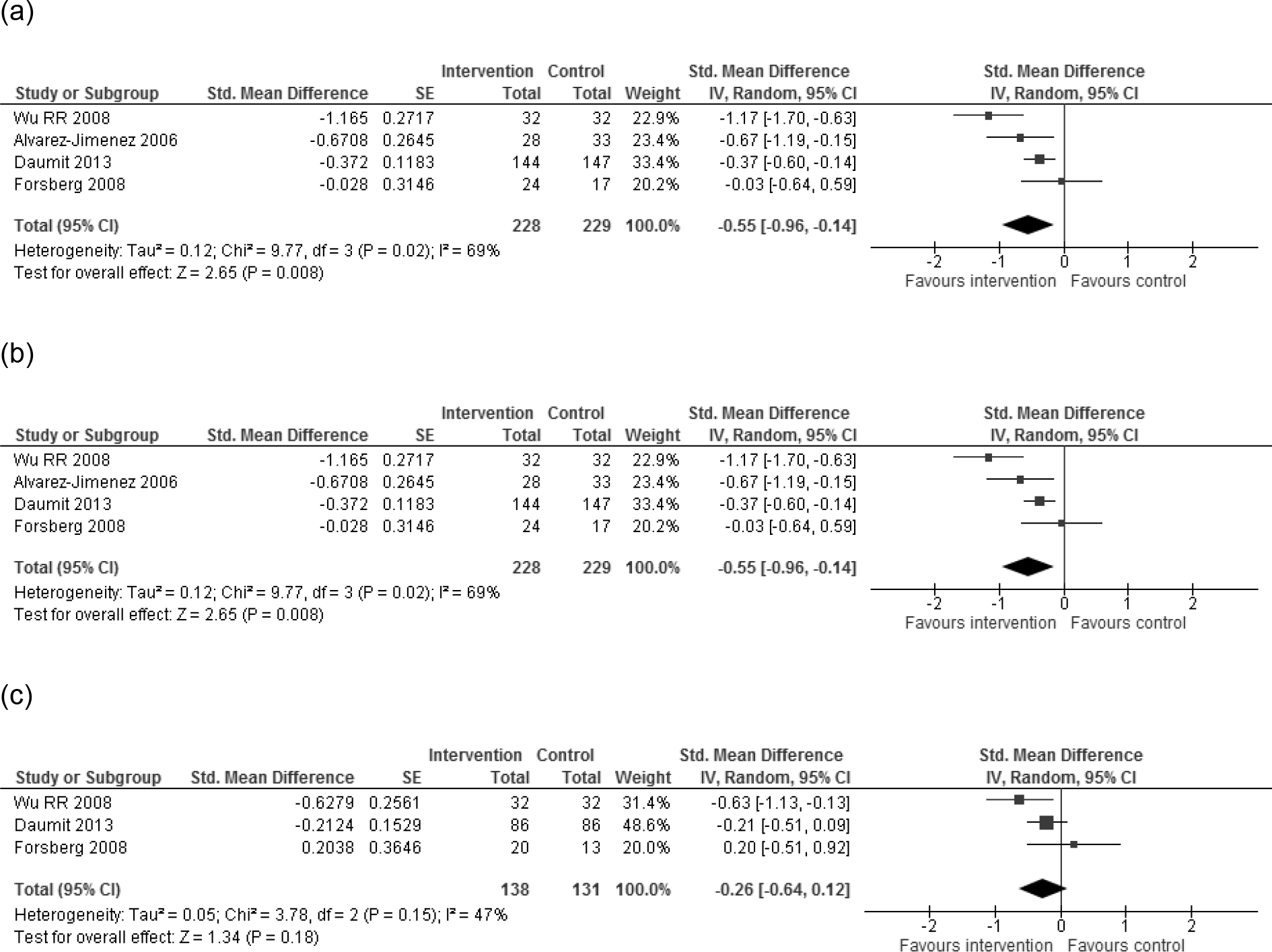

Supplement: Figure S2 — Forest plot a) describes the sensitivity analysis of the effects of lifestyle interventions on body weight. Forest plot b) describes the sensitivity analysis of the effects of lifestyle interventions on waist circumference. Forest plot c) describes the sensitivity analysis of the effects of lifestyle interventions on insulin. (TIF) [file pone.0112276.s002.tif]

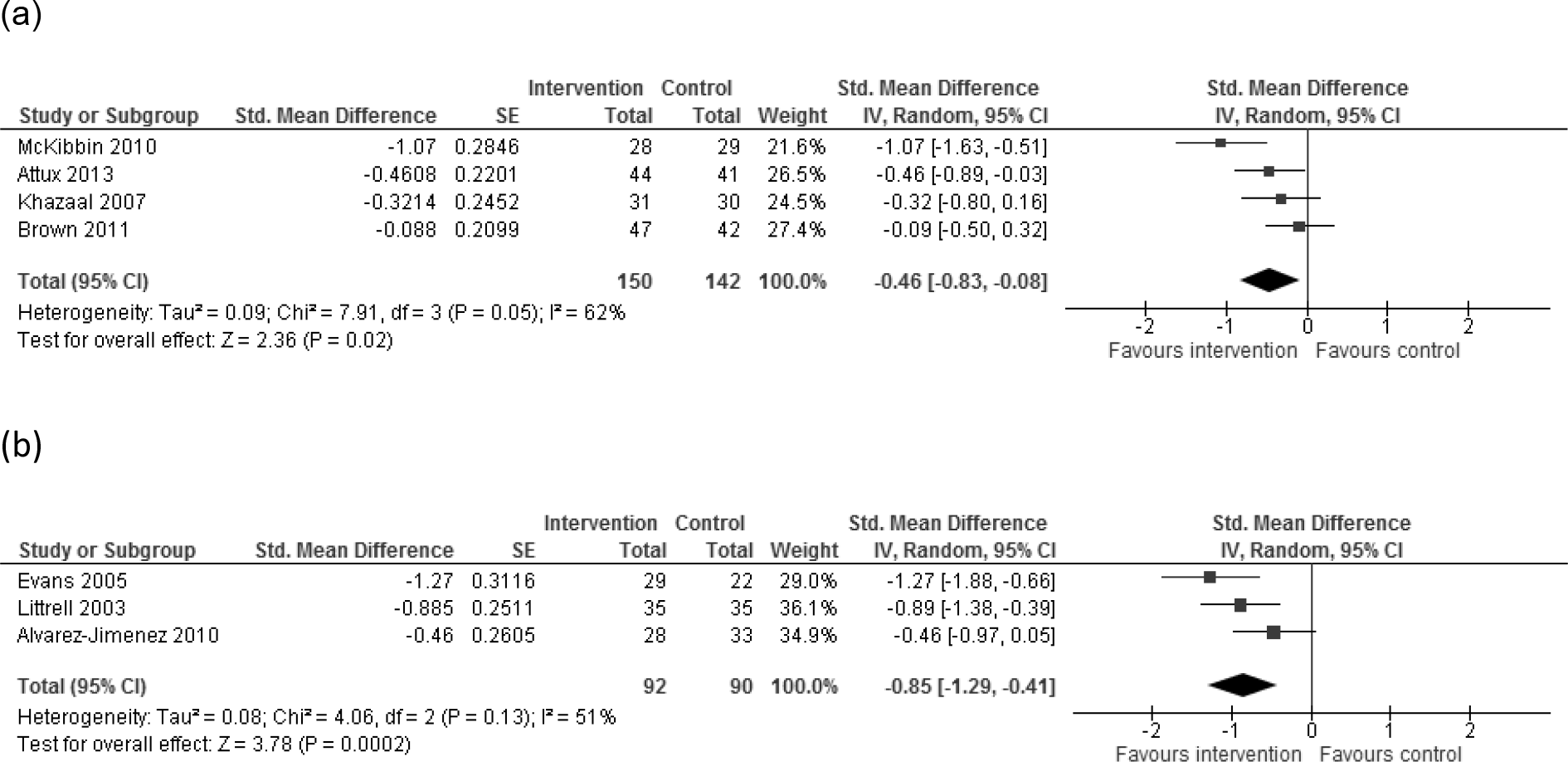

Supplement: Figure S3 — Forest plot a) describes the longterm follow-up effects of weight loss interventions on bodyweight. Forest plot b) describes the longterm follow-up effects of weight gain prevention interventions on bodyweight. (TIF) [file pone.0112276.s003.tif]

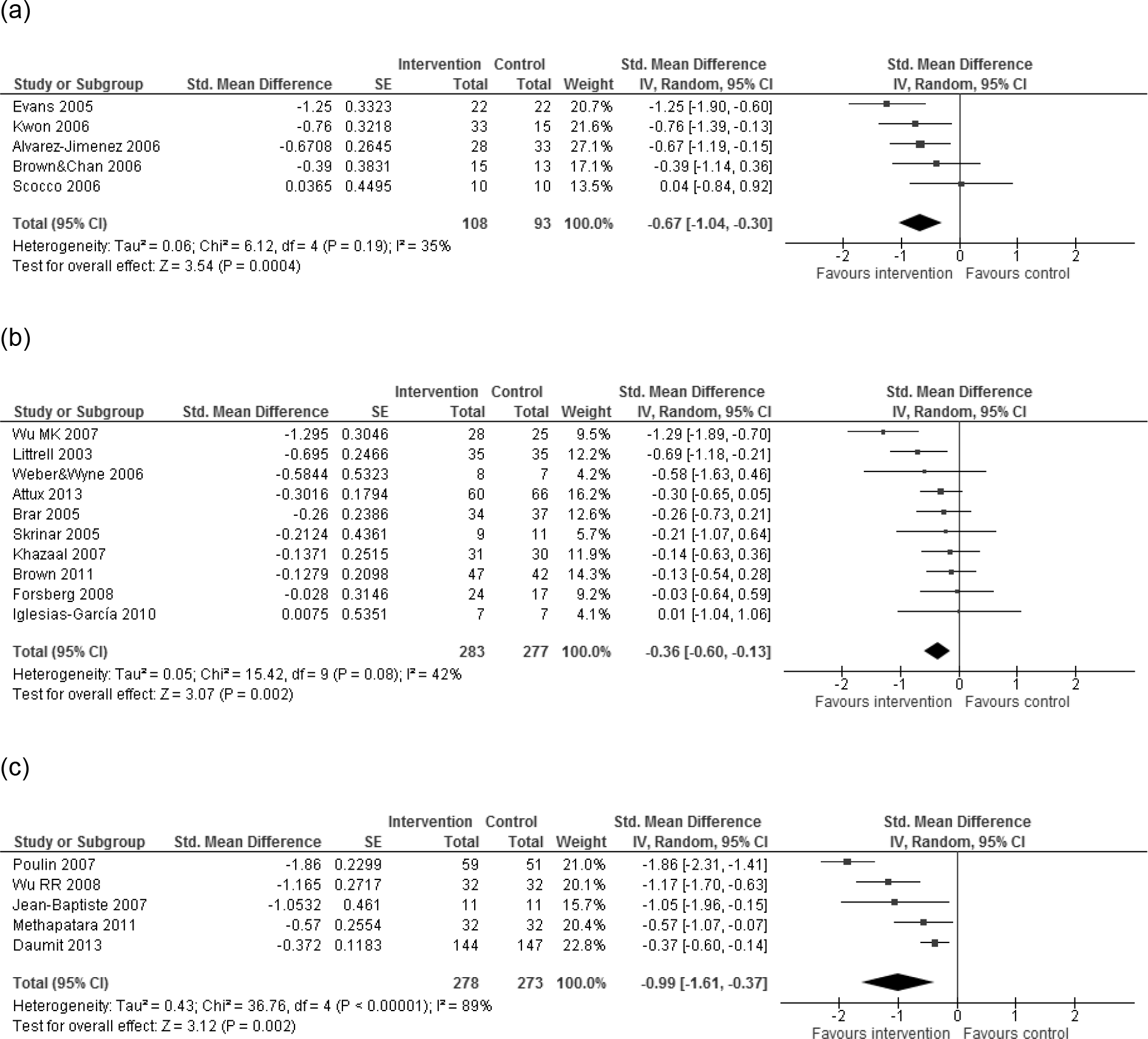

Supplement: Figure S4 — Forest plot a) describes the effects of individual interventions. Forest plot b) describes the effects of group interventions. Forest plot c) describes the effects of combined interventions. (TIF) [file pone.0112276.s004.tif]
